# Anti-inflammatory approaches in cancer immunotherapy-induced autoimmunity

**DOI:** 10.1016/j.ero.2026.100223

**Published:** 2026-08-08

**Authors:** Robert Zeiser

**Affiliations:** Department of Medicine I, Medical Center - University of Freiburg, Faculty of Medicine, University of Freiburg, Freiburg, Germany

## Abstract

Cancer immunotherapy, including allogeneic haematopoietic cell transplantation, infusion of chimeric antigen receptor T (CAR-T) cells, bispecific antibodies, and immune checkpoint inhibitors (ICIs), has revolutionised the treatment of cancer. However, therapy resistance and immune-mediated side effects reduce the overall success. Recent developments in these 2 areas were reported at the 2026 ATT conference. Treatment of immune effector cell-associated neurotoxicity syndrome (ICANS) using transforming growth factor β (TGFβ)-activated kinase-1 (TAK1)-NF-κB-p38 mitogen-activated protein kinase (MAPK) pathway inhibition was shown to be effective in preclinical models. TAK1 inhibition reduced microglia activation and improved neurocognitive function after CAR19 T-cell transfer. This review discusses new developments in the fields of ICANS, graft-vs-host disease (GVHD), and ICI-mediated side effects.

Allogeneic haematopoietic cell transplantation, infusion of chimeric antigen receptor T (CAR-T) cells, bispecific antibodies, and immune checkpoint inhibitors (ICIs) have revolutionised the treatment of cancer. However, therapy resistance and immune-mediated side effects reduce the overall success. Recent developments in these 2 areas were reported at the 2026 ATT conference. Treatment of immune effector cell-associated neurotoxicity syndrome (ICANS) using transforming growth factor β (TGFβ)-activated kinase-1 (TAK1)-NF-κB-p38 mitogen-activated protein kinase (MAPK) pathway inhibition was shown to be effective in preclinical models [1]. TAK1 inhibition reduced microglia activation and improved neurocognitive function after CAR19 T-cell transfer [1].

ICI-induced colitis and hepatitis that do not respond to corticosteroids are currently treated without any US Food and Drug Administration (FDA)/European Medicines Agency (EMA)-approved drugs. It was recently shown that ICI colitis responds to extracorporeal photopheresis (ECP) without blocking antitumour immune responses in mice [2]. The antitumour effect of ICI treatment was maintained in mice after ECP, while it was reduced with glucocorticosteroids, α4β7-integrin blockade, and tumour necrosis factor α (TNFα) inhibition [2]. ECP induced adiponectin production in the inflamed tissue, which reduced proinflammatory tissue-resident T-cell frequencies in the human colon [2]. A prospective phase 1b/2 trial including 14 patients with ICI-mediated side effects led to overall response rates of 92%, whereas ECP-related toxicity was low [2].

Allogeneic haematopoietic cell transplantation (allo-HCT) is a potentially curative therapy for a variety of conditions, including haematologic disorders, metabolic storage diseases, immune deficiencies, and haematological malignancies. Relapse of acute myeloid leukaemia (AML) and graft-vs-host disease (GVHD) are major hurdles that need to be overcome to make allo-HCT safer [3]. The classical prevention and treatment of GVHD includes different immunosuppressive approaches such as calcineurin inhibitors, antimetabolites, mammalian target of rapamycin (mTOR) inhibitors, corticosteroids, and others [3]. Novel strategies to reduce immunosuppressive therapy after allo-HCT include regenerative approaches such as keratinocyte growth factor, interleukin (IL)-7, R-spondin, pentagastrin, or glucagon-like peptide-2 (GLP-2). Such tissue regenerative approaches act on the intestinal tract by their protective impact on Paneth cells and intestinal stem cells. At ATT 2026, novel data on the role of gastrin in GVHD was discussed. Gastrin was shown to induce intestinal regeneration while sparing the graft-vs-leukaemia (GVL) effect [4]. Intestinal L cells produce GLP-2, which is an enteroendocrine tissue hormone that can cause tissue regeneration. In mice and patients developing GVHD, intestinal GLP-2 levels were found to be decreased [5]. Treatment with the GLP-2 agonist, teduglutide, reduced de novo acute GVHD (aGVHD) and steroid-refractory GVHD [5]. Another central concept for aGVHD treatment is JAK1/2 inhibition with ruxolitinib, which was successfully translated from the mouse model into a clinical application and ultimately FDA/EMA approval [6]. The next step after the successful phase 3 trials may be a combination—eg, the combination of ruxolitinib with ECP was shown to have activity on chronic GVHD. Novel approaches in GVHD could include kinase inhibitors for kinases relevant for the disease. GVHD caused activation of microglia cells, as shown by morphological changes and differential gene expression. Selective genetic ablation of TAK1 or TNF in microglia identified the TAK1/TNF/major histocompatibility complex II (MHC-II) axis as a central mediator of central nervous system (CNS) inflammation. The kinase inhibitor takinib reduced ICANS after CAR-T cell transfer. The role of antibiotics and microbiota in microglial activation during aGVHD of the CNS was recently investigated [7]. It was shown that antibiotic treatment caused infiltration of interferon-γ-producing T cells in the brain, activation of microglia via the TLR4/p38 MAPK pathway, and neurocognitive deficits [7]. The bacteria-derived metabolite N, N, N-trimethyl-5-aminovaleric acid (TMAVA) was shown to decrease upon antibiotic treatment in the brain of mice undergoing allo-HCT [7].

To enhance immune responses against leukaemia without causing GVHD, menin inhibition was tested. Menin inhibition induced transcription of endogenous retroviruses and increased immunogenicity of AML cells *in vitro* and *in vivo* [8]. We found that menin inhibition induced class II MHC transactivator (CIITA) and MHC-II expression in Lysine Methyltransferase 2A (KMT2A)-rearranged and Nucleophosmin 1 (NPM1)-mutated AML cells *in vitro* and *in vivo* [8]. Increased MHC-II expression sensitised AML cells to T-cell–mediated cytotoxicity in mice. Menin inhibition enhanced the GVL effect in leukaemia mouse models [8].

The findings presented at ATT 2026 indicate that novel immunoregulatory approaches have yielded promising results in preclinical studies and early clinical trials.

## CRediT authorship contribution statement

**Robert Zeiser:** Conceptualization, Formal analysis, Funding acquisition, Supervision, Writing – original draft, Writing – review & editing.

## Competing interests

RZ reports was provided by University Medical Center Freiburg. RZ reports a relationship with Novartis Pharmaceuticals Corporation that includes: speaking and lecture fees.
